# Supplementary material for: CRISPR/Cas9 Screenings Reveal the Role of STX1A and CDK1 in Cathepsin G Entering and Killing Colorectal Cancer Cells
Source: Interdiscip Inf Sci. Author manuscript; Available in PMC 2026 Apr 2. (PMC13042082; doi:10.4036/iis.2025.A.11)
Supplement: Supplementary Material [file NIHMS2157563-supplement-Supplementary_Material.pdf]

Supplemental materials

Supplemental figures

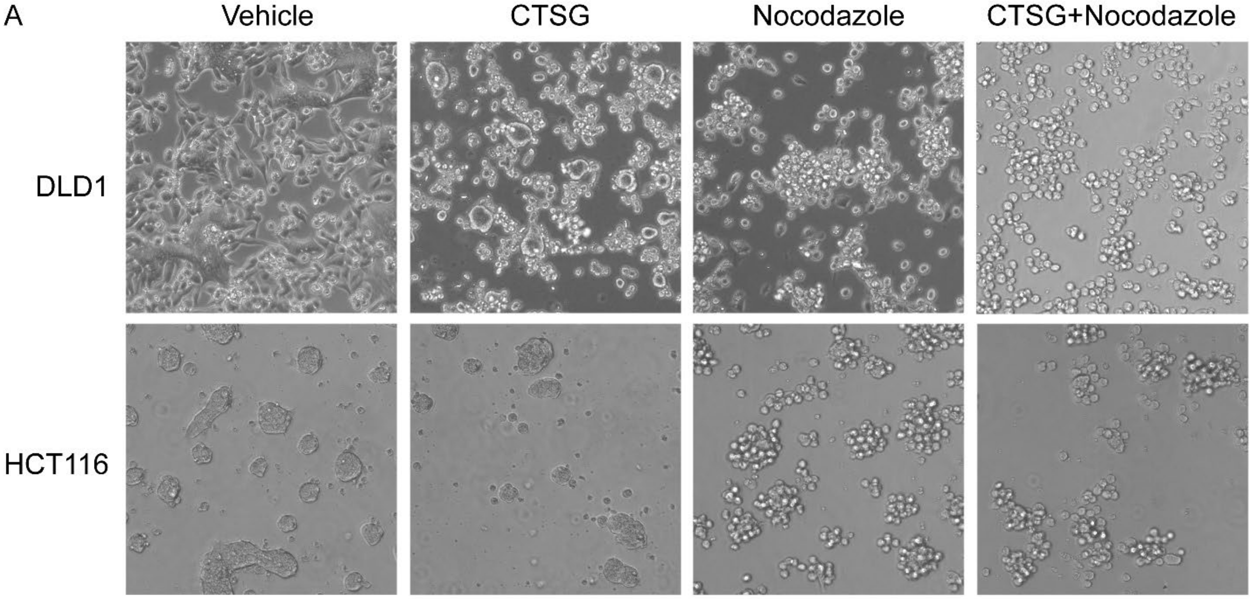

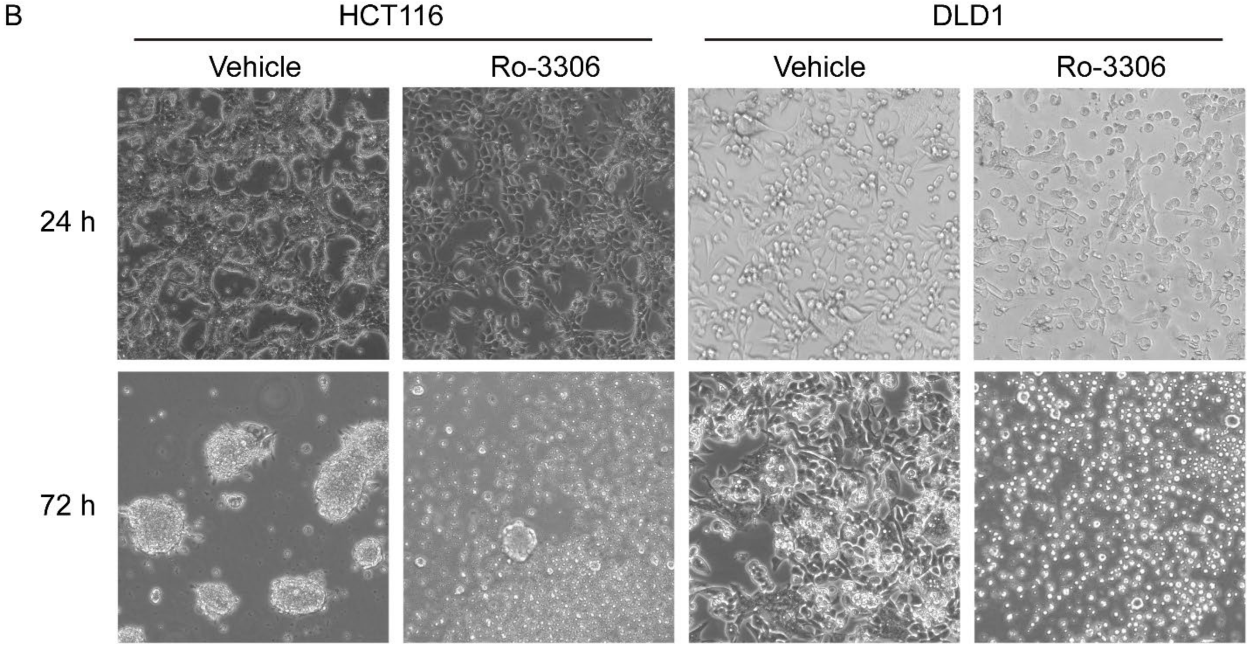

Supplementary Figure 1. CDK1 inhibitor attenuates the killing function of CTSG in a cell cycle-independent manner. (A) HCT116 and DLD1 cells were treated with 5 ng/μL of CTSG, 1 ng/μL of nocodazole, or their combination for 24 hours. The data presented are representative images of cellular morphology. (B) HCT116 and DLD1 cells were treated with vehicle or 10 μM Ro-3306 for 24 or 72 hours. The data presented are representative images of cellular morphology.

Supplemental tables

Supplementary table 1. 141 human membrane trafficking genes are included in the arrayed sgRNA library.

Target order	Gene Symbol	Target order	Gene Symbol	Target order	Gene Symbol
1	AP1G2	51	ENG	101	STX5
2	AP2A1	52	GBF1	102	STX6
3	AP2M1	53	HPS5	103	STX7
4	AP3D1	54	HSPA8	104	STXBP1
5	ARHGAP33	55	IQSEC1	105	STXBP2
6	CACNA1D	56	SNAP25	106	SYT10
7	CACNA1F	57	SNAP29	107	SYT15
8	AP1G1	58	STX12	108	SYT4
9	AP1M1	59	STX18	109	SYT5
10	AP1M2	60	STX4	110	SYT9
11	AP1S1	61	STX8	111	VAMP1
12	CACNA1B	62	STXBP5	112	VAMP3
13	CACNA1C	63	STXBP6	113	VAMP4
14	CAV2	64	SYT1	114	VAMP8
15	DNM1	65	SYT13	115	VPS13B
16	EHD1	66	SYT16	116	VPS33B
17	EHD4	67	SYT17	117	ESYT2
18	ESYT3	68	SYT2	118	STXBP3
19	HPS4	69	SYT3	119	STXBP4
20	HUWE1	70	SYT6	120	AP1AR
21	PLIN3	71	SYT7	121	AP1S2
22	PREB	72	SYT8	122	AP3S2
23	STX11	73	VAPB	123	AP4S1
24	STX1A	74	AP1S3	124	ARHGAP32
25	STX1B	75	AP2A2	125	CACNA1H
26	SYNRG	76	AP3B2	126	CDKN2A
27	SYT11	77	AP3M1	127	CLTC
28	AP1B1	78	CACNA1E	128	COPB1
29	AP2B1	79	CACNA1I	129	COPG2
30	AP2S1	80	CACNA1S	130	COPZ2
31	AP3B1	81	CAV1	131	HPS3
32	AP3M2	82	COPE	132	HPS6
33	AP3S1	83	COPZ1	133	LYST
34	AP4B1	84	EHD3	134	SAR1A
35	AP4E1	85	HPS1	135	SEC24B
36	AP4M1	86	ITGA3	136	STX17
37	ARF1	87	LMAN1	137	SYT12
38	ARHGAP10	88	PTGES2	138	SYT14

39	CACNA1A	89	RAB3A	139	TBC1D15
40	CACNA1G	90	RACGAP1	140	VAMP2
41	CAV3	91	RASA1	141	VAMP7
42	CLTA	92	RGS6		
43	CLTB	93	SAR1B		
44	COPA	94	SEC23A		
45	COPB2	95	SEC31A		
46	COPG1	96	SNAP23		
47	DNM2	97	STX10		
48	DNM3	98	STX19		
49	DTNBP1	99	STX2		
50	EHD2	100	STX3		

Supplementary table 2. 40 membrane trafficking genes, knocking out which relieved CTSG induced upregulation of cleaved PARP to a level stronger than knocking out RAGE.

Gene symble	Fold change	log2(Fold change)
PREB	0.43	-1.23
CACNA1A	0.50	-1.00
RAB3A	0.61	-0.72
CACNA1B	0.63	-0.68
STX11	0.64	-0.64
STX1A	0.72	-0.48
SYT3	0.72	-0.47
ESYT3	0.73	-0.46
HPS4	0.74	-0.43
IQSEC1	0.79	-0.34
STXBP5	0.84	-0.25
AP3M2	0.84	-0.24
STX12	0.90	-0.16
STX3	0.90	-0.15
AP3M1	0.92	-0.12
RACGAP1	0.92	-0.11
HUWE1	0.97	-0.05
SYT4	0.98	-0.03
AP1G2	0.98	-0.02
STXBP1	0.99	-0.02
SEC23A	0.99	-0.02
STXBP6	0.99	-0.01
AP3D1	1.00	-0.01
AP3S1	1.03	0.04
CACNA1F	1.04	0.05
EHD4	1.04	0.05
SYT10	1.07	0.09
SYT13	1.07	0.09
STX19	1.08	0.11
COPE	1.08	0.11
AP4M1	1.08	0.11
HPS5	1.09	0.12
AP2B1	1.11	0.14
SYT17	1.12	0.16
SYT5	1.12	0.16
AP1B1	1.12	0.17
AP1S1	1.13	0.17
SYT15	1.15	0.20
SYNRG	1.16	0.21
CDKN2A	1.18	0.24

Supplementary table 3. Top 10 increased and decreased genes in neutrophil conditional medium treated DLD1 cells compared to fresh medium cultured DLD1 cells.

Gene	sgRNA	CM-N|beta	CM-N|z	CM-N|p-value
PSMB7	4	0.68592	0.90644	0
TMEM88B	4	0.5362	1.0924	5.23E-05
NDC80	4	0.50108	0.79144	0.00010463
DEFB106B	2	0.50077	0.76842	0.0044468
RSRC1	4	0.49446	1.0511	0.00015694
NRF1	4	0.49007	1.1138	0.00015694
SCD	4	0.48661	0.88939	0.00015694
RBM22	4	0.47152	0.69873	0.00015694
CDK1	4	0.46026	0.87898	0.00015694
ISY1-RAB43	4	0.44773	1.1895	0.00015694
KDELR2	4	-0.43261	-1.1169	0.0003662
INCENP	4	-0.43795	-1.2528	0.0003662
POLR2A	4	-0.44258	-1.2716	0.0003662
SETD8	4	-0.45891	-1.6657	0.00026157
DROSHA	4	-0.46408	-1.9457	0.00026157
RPS15	4	-0.48098	-1.7574	0.00026157
SNRPD3	4	-0.51662	-1.6884	0.00026157
WASH1	4	-0.51714	-2.0189	0.00026157
SNRPA1	4	-0.5493	-1.424	0.00020926
ZNF830	4	-0.61166	-2.0198	0.00010463
